# Improving Pedestrian Safety Using Ultra-Wideband Sensors: A Study of Time-to-Collision Estimation

**DOI:** 10.3390/s23084171

**Published:** 2023-04-21

**Authors:** Salah Fakhoury, Karim Ismail

**Affiliations:** Department of Civil and Environmental Engineering, Carleton University, 1125 Colonel By Dr, Ottawa, ON K1S 5B6, Canada; salah.fakhoury@carleton.ca

**Keywords:** pedestrian safety, time-to-collision (*TTC*), ultra-wideband (UWB), wireless sensors, UWB beacons

## Abstract

Pedestrian safety has been evaluated based on the mean number of pedestrian-involved collisions. Traffic conflicts have been used as a data source to supplement collision data because of their higher frequency and lower damage. Currently, the main source of traffic conflict observation is through video cameras that can efficiently gather rich data but can be limited by weather and lighting conditions. The utilization of wireless sensors to gather traffic conflict data can augment video sensors because of their robustness to adverse weather conditions and poor illumination. This study presents a prototype of a safety assessment system that utilizes ultra-wideband wireless sensors to detect traffic conflicts. A customized variant of time-to-collision is used to detect conflicts at different severity thresholds. Field trials are conducted using vehicle-mounted beacons and a phone to simulate sensors on vehicles and smart devices on pedestrians. Proximity measures are calculated in real-time to alert smartphones and prevent collisions, even in adverse weather conditions. Validation is conducted to assess the accuracy of time-to-collision measurements at various distances from the phone. Several limitations are identified and discussed, along with recommendations for improvement and lessons learned for future research and development.

## 1. Introduction

### 1.1. Background

Wireless signals emitted from devices commercially available to consumers operate at various frequencies and signal modes. One type of the signal modes that is growing in popularity is the ultra-wideband (UWB). UWB wireless communication technology allows high-frequency signal transmission with relatively low power consumption. UWB is a radio frequency technology that uses a large bandwidth with low power to transmit data over short distances. UWB frequencies range from 3.1 to 10.6 GHz and have greater bandwidth than alternative wireless technologies, including Wi-Fi and Bluetooth [1]. A device that functions as a source of UWB wireless signal emission is often called a UWB beacon. UWB beacons play an essential role in modern network-based applications, such as the Internet of Things (IoT), by delivering location-based services through a wireless communication protocol. Many applications have been developed based on UWB sensors, including proximity sensing and tracking, to improve the functionality of IoT devices. UWB is often used to precisely determine the position of beacons relative to a receiver without interfering with other wireless technology. The UWB communication module consumes relatively little power and is inexpensive compared to other wireless sensors. UWB chips have been included in some recent designs of smartphones, e.g., Apple’s iPhone 11 and later models. For the purpose of positioning a wireless signal source, UWB offers one of the most accurate solutions for distance measurement between two communicating devices using the time-of-flight technique. Therefore, UWB is adopted in this work as the main wireless mode to develop the proposed safety application as well as detect safety-relevant events.

Wireless communication using UWB is adopted in many applications, such as asset tracking, indoor navigation, and autonomous vehicles. In the field of transportation, UWB can play a role by allowing effective navigation and communication. By virtue of their precise distance measurement, UWB beacons have the potential to provide real-time information about potential hazards on the road. The extraction of road user proximity indicators can also help in identifying safety shortcomings at a cross-sectional or site-specific level. This can support traffic safety applications that aim at utilizing non-collision and safety-relevant data. For instance, UWB can enable real-time vehicle-to-vehicle communication by sharing positional information, such as range, relative speed, and direction [2]. Another potential safety application is that UWB can provide highly accurate positioning information to enhance navigation, thus reducing the risk of a collision caused by incorrect navigation. Finally, UWB technology can enhance pedestrian safety by detecting and tracking the movement of pedestrians or other vulnerable road users, allowing vehicles to react promptly to potential hazards. Conversely, vehicles can track pedestrian positions and movements and, if appropriate, alert the pedestrian to an impending hazard. Therefore, UWB technology has an excellent opportunity to enhance transportation safety. 

### 1.2. Literature Review

Extensive research has been conducted to analyze the use of wireless sensors, such as Bluetooth, Bluetooth Low Energy (BLE), Wi-Fi, UWB, or GPS, in real-time traffic monitoring applications [3,4,5,6]. Traffic monitoring applications are designed to provide real-time information about traffic conditions; for instance, the calculation of surrogate measures of safety to estimate the risk of collision or the severity of traffic events that are not collisions themselves [7]. Researchers also explored the use of smartphones to alert drivers of a potential accident in the case of a vehicle-to-vehicle interaction [8]. For example, the real-time application warns drivers by combining information from traffic conflict indicators, e.g., the time-to-collision and deceleration rate to avoid a crash, and the use of satellite navigation systems. This application can create a safety map that provides awareness to drivers of high-risk areas and when they are approaching “dangerous” zones. They claimed that the real-time warning system suffers from errors in GPS measurements [8].

A recent study was conducted to evaluate UWB smartphones from major manufacturers, such as Samsung, Apple, and Google [9]. The research found that the tested devices could measure distances with an error of under 20 cm. However, the devices did not provide consistent measurements in outdoor, lab, and garage scenarios. The maximum range distances possible to measure with UWB-enabled smartphones were 40 m, 23 m, and 11.6 m for the iPhone 12 Pro, Samsung Galaxy S21 Ultra, and Google Pixel 6 Pro, respectively. The study showed that the iPhone and Google Pixel demonstrated some measurement errors (distance reduction) of up to 3 m at a range distance of 5 m because of the potential multipath environment even, in an outdoor environment.

In another study, researchers created a smartphone application to warn distracted pedestrians while crossing [10]. The research utilized Bluetooth beacons around a signalized intersection to alert pedestrians who were distracted by their phones when they were getting close to a dangerous intersection, either through a visual or audible warning. The application was tested in a real-life deployment to increase public safety in urban environments. Another study [11] focused on the use of Bluetooth Low Energy (BLE) beacons and smartphones to provide location-based and proximity-based services for a smart parking application. A BLE beacon is a small wireless device that uses low-power Bluetooth technology to communicate with nearby devices. The experiments were conducted outdoors and indoors to evaluate proximity accuracy. The evaluation was achieved by analyzing the Received Signal Strength Indicator (RSSI). BLE beacons were used to fill the gaps in GPS data since the GPS receiver cannot perform adequately in some indoor locations.

Many studies looked at utilizing wireless sensors in traffic safety in order to enhance pedestrian safety. For instance, one study investigated the use of wireless sensors, such as Bluetooth, Wi-Fi, and BLE, in vehicle–pedestrian collision warning systems [12]. Their study conducted field experiments to compare the performance of each mode. Moreover, five factors, including the received signal strength indicator (RSSI)-based distance relationship, motion effects, rainfall effects, signal transmission rates, and non-line-of-sight effects, were evaluated. The study found that BLE mode was superior to Bluetooth and Wi-Fi modes because it showed better accuracy in estimating distance and position. Another study [4] conducted an experiment to look at the use of Wi-Fi technology in traffic data collection. Wi-Fi technology has been used in vehicle and pedestrian positioning in various applications, but the technology faces challenges that have impeded tracking, such as Media Access Control Address (MAC) randomization.

The Federal Highway Administration (FHWA) funded PedPal, a mobile application that was created by [13,14] to help pedestrians with disabilities conduct safe crossings at signalized intersections. The real-time localization was obtained by using stationary UWB and Bluetooth beacons at each corner of an intersection. Moreover, the study looked at enhancing pedestrian safety by considering accessible route navigation and planning, safe intersection crossings, and pedestrian-to-infrastructure communication. For instance, when a pedestrian is approaching an intersection, the beacons start communicating with the smartphone. The position of a pedestrian can be assumed by their proximity to a beacon. The crossing direction can be tracked by the distances obtained from all beacons. This study found that utilizing UWB beacons allowed for better accuracy than Bluetooth beacons. Finally, the study showed that UWB beacons could be used to overcome the errors of Bluetooth beacons. It was noted that UWB technology showed high accuracy in localization and tracking even in extreme weather conditions [15]. For instance, the study looked at enhancing cyclist safety by placing UWB tags on bikes and vehicles and placing UWB anchors at a signalized intersection.

### 1.3. Study Objectives

This study aims to explore the potential to improve pedestrian safety using UWB technology. The specific objectives of the study are as follows: [i] develop a prototype that can detect traffic conflicts based on severity using UWB sensors, [ii] assess the performance of this system using various statistical measures, [iii] evaluate the impact of errors in distance measurements on the system’s performance, and [vi] document lessons learned for future technology development. The following sections present: technology description, field experiments, discussion, and conclusions, respectively.

## 2. Materials and Methods

UWB beacons transmit data over a large bandwidth, unlike traditional Bluetooth beacons. UWB beacons allow high accuracy in measuring distances and tracking positions between the device and beacon. Beacons can connect to nearby devices that are UWB-enabled or, in the case of smart devices, Apple devices equipped with a U1 chip. For example, the phone used in this study was the iPhone 13 Pro Max, which has an embedded U1 chip that can communicate with UWB beacons. When a phone and a UWB beacon connect, the distance measurement can be calculated by the time-of-flight and can yield distance measurements with, as is often claimed, inch-level precision. Therefore, due to these attributes, UWB beacons are more suitable for service and real-time location applications than BLE beacons.

The UWB beacons used in this study were purchased in 2022. The development kit includes UWB beacons and a software development toolkit (iOS SDK), through which a developer can access real-time distance measurements between the beacon and a U1-equipped iPhone. With access to distance measurements, a dedicated spatially aware application was developed that utilizes sequences of distance measurements to calculate the relative speed and acceleration of the beacon with respect to the phone. Furthermore, safety-relevant measures can be calculated to estimate the hazard that a pedestrian holding this phone is exposed to from an approaching vehicle equipped with a beacon.

### 2.1. Distance Measurement Accuracy

An exploratory study was conducted to assess the real-world accuracy of distance measurements for stationary (non-moving) beacons. This is an initial assessment in a highly controlled environment because dynamic distance measurements, which are introduced later, involve more experimental set-up variables and less controllable environments. Stationary UWB beacon experiments were conducted to evaluate the performance of a UWB beacon in an outdoor and indoor environment. This study tested the UWB beacons purchased from Estimote (as mentioned earlier) due to their compatibility with U1-equipped iPhones. The experiment looked at the range, accuracy, and reliability of UWB beacons to communicate with an iPhone 13 Pro Max. The true distances between the smartphone and stationary UWB beacon were set up as follows: 1 m, 3 m, 10 m, 15 m, 20 m, and 25 m. Range data was gathered for a duration of 1 minute at each distance in order to obtain stable estimates and gather a representative sample of measurements at each location. This is because, on average, there are three range measurements every second. It is noteworthy that this experiment was intended to gauge the accuracy of distance measurements for a stationary beacon, which is the backbone for further analysis involving a moving beacon.

The distance between the phone and beacon was recorded using a dedicated application provided by the manufacturer. It is noteworthy that the iOS safety application developed as part of this study (to be presented later) was developed using the software development kit based on this basic application. This application displays the serial numbers of the nearby beacons (fingerprints) and the reported distance between each beacon and the phone running this application. All measurement data were uploaded to an online database to enable time-extended recording of data and conduct more sophisticated analysis. Table 1 demonstrates a sample of raw data from the outdoor experiment and shows that the detection rate is approximately every 350 ms. However, in the indoor experiment, the detection rate could fall to every millisecond in some instances, which caused repeated values and inaccurate speed measurements. Therefore, a condition was later applied to discard any detections with a timelapse of less than 100 ms. The outdoor and indoor experiment data were collected and analyzed to evaluate the beacon’s performance and identify areas for improvement.

The outdoor experiment was conducted on December 2022, at an ambient air temperature of −9 °C. It was found that the application ceased to work properly when the phone was exposed to cold weather for more than 15 minutes. The experiment was occasionally paused to warm up the phone and then resumed. As for the indoor environment, in which the ambient air temperature was 13°C, the results were much more consistent. The results of stationary UWB beacon experiments are shown in Table 2, which compares the actual distances and estimated UWB beacon distances. At a 1.00 m distance, the Mean Average Percentage Error (MAPE) was 10.93%, and the average MAPE from all experiments was 2%. Furthermore, the number of detections per minute ranges from 178 to 197 detections for 1.00 m to 20.00 m distances, respectively. However, at a distance of 25.00 m, the number of detections per minute dropped to 49. The reliable range of detection was determined to be up to 20.00 m, but the signal can be detected at 25.00 m. Therefore, the results can be used to inform the development of new services and applications to improve the performance of UWB beacons and deliver best practices.

### 2.2. Development of a Safety Assessment Protocol for Pedestrian–Vehicle Interactions

A safety application was developed to measure the hazard that an approaching vehicle presents to a pedestrian carrying a smartphone. An appropriate proximity measure needs to be calculated to represent this hazard. The proximity measure chosen is time-to-collision (*TTC*), which represents the time that will elapse from the current moment until an approaching vehicle collides with the smartphone (assuming the movement of the approaching vehicle remains unchanged). The safety application can estimate the speed and acceleration of the approaching vehicle. This can also enable the calculation of *TTC* that takes into consideration vehicle acceleration.

*TTC* is one of many conflict indicators used to characterize the safety of a traffic conflict but remains one of the most common indicators [16]. Many studies analyzed crash data to identify potential factors affecting pedestrian safety. However, analyzing pedestrian safety using crash data is challenging on many accounts. First, pedestrian-involved crashes are rare yet catastrophic. Second, relying on pedestrian crashes to measure safety is a reactive approach that requires a collision to occur before a safety assessment is performed. For that purpose, surrogate measures of safety, e.g., *TTC*, are utilized to assess the safety of relevant traffic events that happen more frequently than crashes but do not involve a crash themselves. According to a previous study [17], many studies rely on surrogate measures of safety to investigate pedestrian–vehicle interactions. A traffic conflict is conceptually defined in the literature as follows: “an observable situation in which two or more road users approach each other in space and time to such an extent that there is a risk of collision if their movements remain unchanged.” In that case, when two road users have a reportable *TTC*, then the collision is expected to occur. The imminence of such a collision is inversely proportional to *TTC*, with small values indicating heightened severity and hazard level.

Traffic conflict can happen at various locations, including crosswalks, intersections, and roundabouts. Furthermore, *TTC* is used to evaluate the risk of a collision between a vehicle and a pedestrian or between two interacting vehicles. From the driver’s perspective, *TTC* information is used in advanced driver assistance systems, including collision avoidance systems, to give drivers collision warnings and potentially prevent accidents. Moreover, *TTC* information can assist vehicles in making decisions regarding their trajectory and speed during autonomous operation. The accuracy of the *TTC* calculation relies on the accuracy of the distance and velocity estimates. Therefore, UWB beacons have great potential for surrogate safety measures and calculating *TTC*. The exact details of *TTC* calculations are presented in later sections.

### 2.3. Time-to-Collision Calculations

The safety application developed in the current study utilizes *TTC* as a traffic conflict indicator in order to assess pedestrian safety. According to a previous study [18], *TTC* measures how much time is left for two road users to crash into each other. *TTC* is classically calculated by estimating the distance and relative velocity between two objects. Usually, knowledge of positions in road safety applications is discrete in time. For example, computer vision detections are performed at a minimum for every frame in a video sequence (approximately 0.03 s). The *TTC* requires the conflicting road users to be on a collision course. Specifically, there is a future position that they are projected to co-occupy if their movements remain unchanged. If the interacting road users are a vehicle and a pedestrian, then the time series of the distances to the earliest collision point can be assumed to be $d_{v}=\left(d_{vt}:t\;\epsilon \;T\right)\;$ and $d_{p}=\left(d_{pt}:t\;\epsilon \;T\right)$, respectively. For simplicity, the time series corresponds to time measurements T that start at the current moment, such that $T=\left(0,\;1,\;2,\cdots \right)$. Generally, *TTC* is the earliest moment between now and when the two road users are projected to come into physical contact. In other words, if the two road users are on a collision course, then the collision will commence at a moment TTC  from now. There are many ways to calculate *TTC*, depending on the assumptions underlying the projections of future positions. The default assumption is that the pedestrian and the vehicle are projected to move at the same velocity until they collide. According to this assumption, during *TTC*, the vehicle will travel distance $d_{v0}$ and the pedestrian will travel distance $d_{p0}$. If the current speeds of the vehicle and the pedestrian can be denoted as $S_{v}$ and $S_{p}$, respectively. This requires independent tracking of both the pedestrian and the vehicle relative to the projected collision point. Some technologies, such as the one used in the current study, can measure the distance between interacting road users. Hence, the projected distance between the pedestrian and the vehicle, $d=\left(d_{t}:t\;\epsilon \;T\right)\;$ is also a time series that can be projected using the current rate of change in the relative distance such that $d_{t}=d_{0}+t\frac{\partial d}{\partial t}{|}_{t=0}$. Because *TTC* is calculable, that is, the two road users are on a collision course, then the following conditions must be satisfied if *TTC* is estimated:

(1) $$\frac{d_{v0}}{S_{v}}=\frac{d_{p0}}{S_{p}}=TTC\;\Rightarrow d_{TTC}=0,$$

where,

(2) $$TTC=d_{0}/\frac{\partial d}{\partial t}{|}_{t=0}$$

Please note that $\frac{\partial d}{\partial t}{|}_{t=0}$ is the relative speed between the two road users measured at the time of estimating *TTC* and is constant (following the assumption that both $S_{v}$ and $S_{p}$ are constant). Both current relative distance and relative speed can readily be obtained using UWB sensors. It is noteworthy that in practice a collision could happen even if the centroids, or a representative point of the road user position, do not co-occupy the same point in space. Specifically, the condition in Equation (1) can be satisfied if $d_{TTC}\leq d_{cr}$, where $d_{cr}$ is some critical spatial proximity between the UWB beacon and phone at which the two road users come into physical contact at other points on their boundaries. The corresponding *TTC* will be $(d_{0}-d_{cr})/\frac{\partial d}{\partial t}$. In the following experiments, the beacon was placed at the front of the vehicle, which directly faces the pedestrian. Hence, for a frontal collision with a crossing pedestrian, $d_{cr}$ is likely a small distance and was therefore ignored in the next sections of this study. 

A key shortcoming of relying on relative distance instead of relative location is that the reverse of the condition in Equation (1) is not always true. That is, when *TTC* is not calculable because the two road users are not on a collision course, it could still be calculable according to Equation (2). In other words, the reliance on the scalar quantity of relative distance and its derivatives of time can produce false positives. To mitigate this issue, in the upcoming experiments, the pedestrian position was assumed to be stationary and offset very closely from the vehicle path. This simulates the case in which the two road users are on a collision course without exposing any of the road users to the real-life hazard of collisions during the experiment. In theory, to eliminate or reduce false positives, multiple beacons must communicate simultaneously with the phone in order to gain more accurate directional information.

The rigid assumption of *TTC* based on constant velocity may not be realistic for accelerating road users. Therefore, another variation of *TTC* is called Modified *TTC*, which accounts for acceleration, speed, and distance for more accurate predictions [19]. Specifically, it utilizes acceleration information to predict how imminent a crash is between two moving road users. When two road users accelerate toward each other, it is expected that Modified *TTC* will be lower than constant-speed *TTC*. When two road users are approaching each other rapidly, the *TTC* value will be lower, so the risk of collision is high. In general, low values of *TTC* are more critical and, in the case of accelerating road users, more realistic. The risk of collision is low when *TTC* is a high value, which indicates the road users are spatiotemporally further separated.

**Figure 1:**
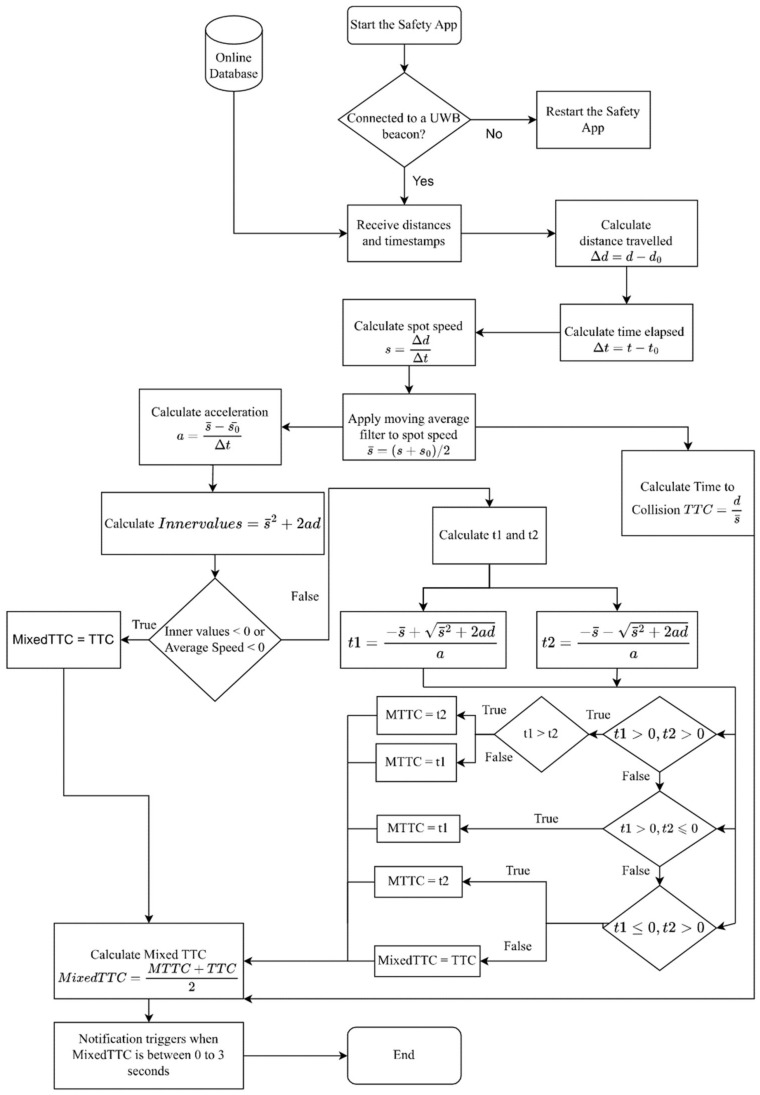
Flowchart showing the Mixed *TTC* calculation procedure.

The proximity measurement in this study was entirely based on distance, relative speed, and relative acceleration. This can be obtained from a single beacon communicating with a phone. The purpose was to trigger an alarm when the approaching vehicle poses a hazard to the phone bearer. Proximity measurement was enhanced through several revisions to calculate speed, acceleration, *TTC*, Modified *TTC*, and a customized variant of *TTC* in the current study called Mixed *TTC*. The collision warning application went through iterative improvement through indoor trials and early experiments. Key improvements focused on robust *TTC* measurements. The purpose was to develop an accurate pedestrian safety awareness system to the extent that a pedestrian carrying a smartphone can receive an advance warning of an approaching vehicle in real time. The speed measurement was improved to include a two-step moving average to reduce the sensitivity to momentary errors in distance measurements. The Modified *TTC* was calculated whenever possible but mixed with the *TTC* in order to increase robustness. This was decided after numerous initial trials. The rationale is that Modified *TTC* assumes fixed acceleration, which can be unreasonable for vehicles accelerating from a stop or from a very slow speed. Conversely, the assumption of fixed speed used to support the calculation of simple *TTC* assumes no acceleration whatsoever. This mixture is termed Mixed *TTC* and is intended to represent the arithmetic mean of both measures. Whenever Mixed *TTC* drops below a pre-defined threshold, a hazardous situation is detected. The following flowchart (Figure 1) shows the calculation details that have been used in this application. It is noteworthy that all speeds and accelerations are based on relative distance measurements, i.e., the change in distance between the beacon and phone.

**Figure 2:**
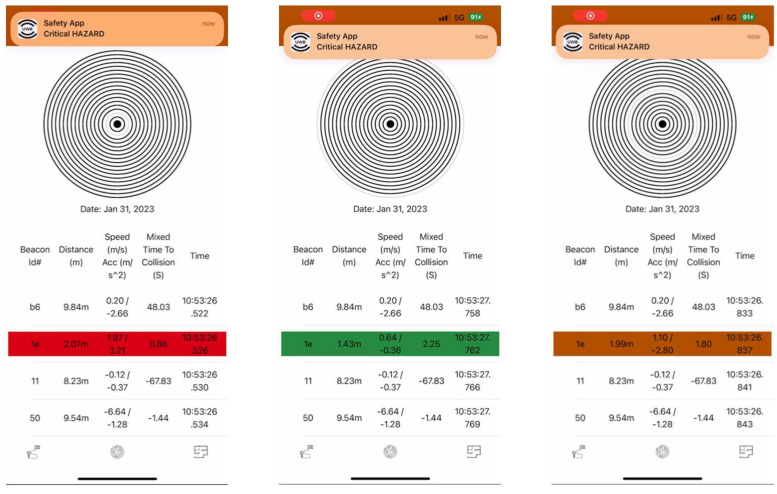
Screenshots of the safety application showing different risk levels for three different hazardous situations.

The safety application was developed to run on Apple’s iOS and is compatible with U1 chip-equipped Apple devices, as shown in Figure 2. For instance, the application presents the detections from all connected UWB beacons. Fingerprints, average speed (speed obtained from a two-step moving average), acceleration, constant-speed *TTC*, Mixed *TTC*, distance, and timestamps are printed on the screen and simultaneously stored on an external online database for real-time as well as offline analysis. The application changes the background color, such as to red, yellow, or green, based on the level of risk. For example, all hazardous Mixed *TTC* values were between 0 and 3 s. When the Mixed *TTC* is between 0 and 1 s, a red background color will appear, representing a high-risk situation. Furthermore, for Mixed *TTC* between 1 and 2 s, the background color changes to yellow, which indicates a medium risk. Finally, when Mixed *TTC* is between 2 and 3 s, the background color changes to green, representing a relatively low risk, as shown in Figure 2. The reader can clearly see the indication at the top of each screenshot labeled “Critical Hazard” as a visual warning to the pedestrian that a vehicle is approaching along a hazardous trajectory and caution is needed.

In order to enhance the collision warning system, a notification is triggered when Mixed *TTC* is in a range of risky values, which is between 0 and 3 s. The notification appears after 100 ms when a risky value is detected and alerts pedestrians of a critical hazard. Furthermore, the notification initiates an emergency sound to help pedestrians recognize the risk of collision and prevent an accident. Finally, the phone will flash, that is, turn on the camera flashlight, to aid in time-stamping the detection. From an experimental perspective, this flash is needed to determine the exact moment a warning was detected from the driver’s view. Many experiments were conducted to evaluate UWB beacons for calculating *TTC* and Mixed *TTC*.

## 3. Results

This collision-warning technology was verified several times in a laboratory environment. Through verification work, many improvements were implemented. Later, it was determined that field performance testing was warranted. The performance testing aims to explore the real-life performance of the developed technology and its potential in assessing vehicle–pedestrian conflict. For the clear visibility of a flashing light and smartphone screen, all testing was conducted under low-light conditions. It is noteworthy that this is a key advantage of wireless technology, as it does not require the smartphone to “see” the approaching vehicle through the smartphone’s rear/front cameras.

### 3.1. Proximity Hazard Detection Experiments

This set of experiments aims to explore the real-time performance of *TTC* calculations. These experiments were conducted to test the accuracy of the UWB beacons in assessing the risk of pedestrian–vehicle conflicts. These experiments were conducted in two different environmental settings: outdoors and indoors (underground parking). The experiments were conducted at different speeds for the approaching vehicle. At each speed, several trials or replications, involving a vehicle approaching the phone location along with dynamic *TTC* calculations, were conducted to ensure consistency of findings. A total of 166 trials were performed in all experimental settings. A failure was defined as a trial in which the approaching vehicle was not able to trigger an alarm on the phone. This could happen for various reasons, including discontinuity in detection or deterioration of the hardware function due to prolonged exposure to cold temperatures. There were a total of 16 failures. Each trial was performed by installing a beacon on an approaching vehicle while an author drove the vehicle at a specific target speed, approaching a tripod-mounted smartphone. The tripod was placed on the sidewalk adjacent to the path of the vehicle such that it is close enough to the vehicle path to calculate *TTC* without precisely occupying the path and resulting in hazardous conditions. The lateral offset of the phone location from the beacon mounted on the vehicle was approximately 1.75 m.

To represent various approaching conditions, the UWB beacon was mounted on a vehicle that approached the smartphone location at various speeds: 3, 5, 10, and 15 km/h, as shown in Table 3. Noted in the table are the trials that were deemed failures. As mentioned earlier, these are trials in which no warning of an approaching vehicle was triggered. Various reasons led to that, but they were mainly due to distance measurement errors. This is discussed in more detail in later sections.

**Figure 3:**
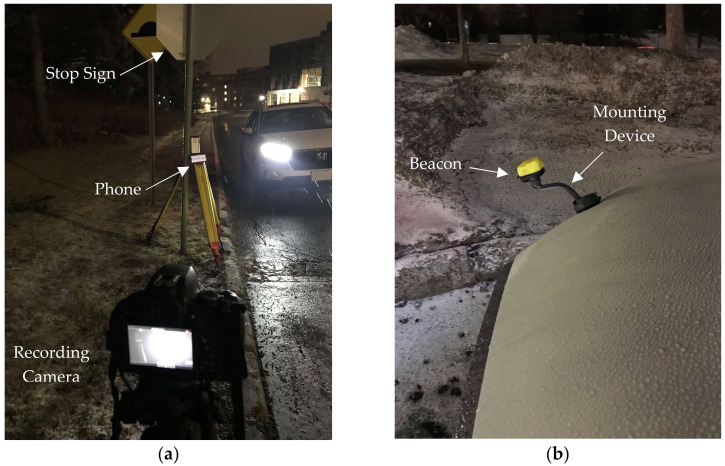
(**a**) The phone mounted on a tripod shows an external recording camera and an approaching vehicle. (**b**) The beacon is mounted on the front right part of the vehicle.

The outdoor experiments were analyzed in relatively harsh winter conditions, such as freezing rain, snow, and cold, to cover many weather conditions. These experiments were conducted on Carleton University’s campus. The outdoor location was at an intersection controlled by a stop sign, and the indoor location was in an underground garage. Figure 3 illustrates the setup of the equipment used in the experiments as well as the beacon location. As guided by previous research [20], the beacon location was at the right and front point of the vehicle, mounted on a specific holder so that the beacon did not rest on the vehicle body, as shown in Figure 3b. Additionally, visible in Figure 3a are the tripods carrying the phone that runs the safety application, as well as another tripod carrying a video camera recording the experiment from the pedestrian’s perspective.

**Figure 4:**
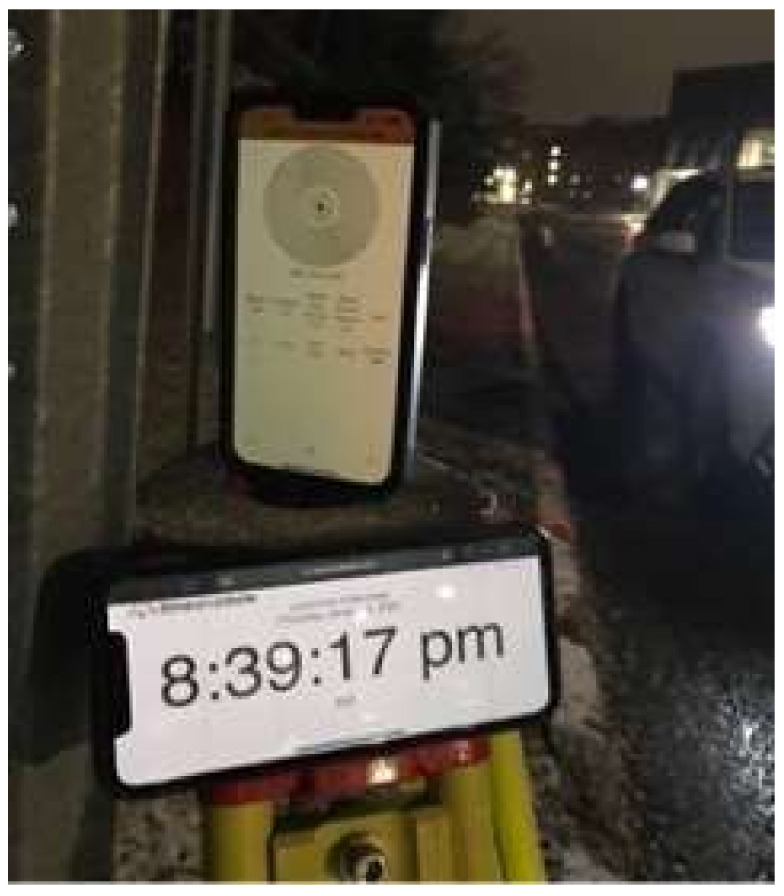
Magnified view of the safety application during the experiment.

Figure 4 shows a magnified image of the phone running the safety application as well as another timing device in order to match the instances from different cameras.

**Figure 5:**
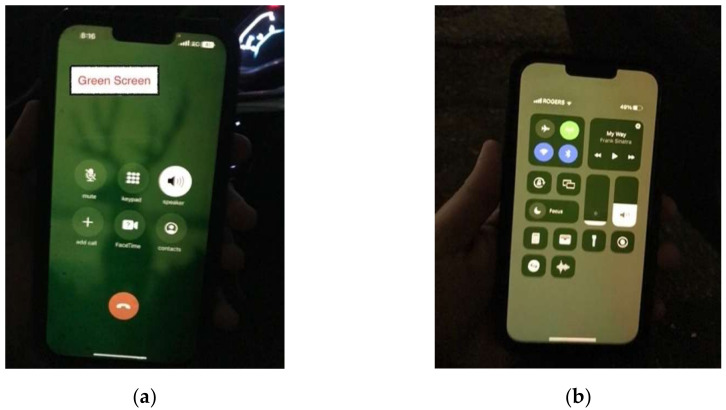
(**a**,**b**) Apparent effect of extended exposure to cold weather on the phone (iPhone 13 Pro Max).

As shown in Table 3, almost all failures occurred outdoors. This led the researchers to hypothesize the impact of cold weather on the performance of the electronics, specifically the UWB chip in the phone. A previous study [21] discussed the effect of cold weather on smartphones and noted how their performance was impacted. In January 2023, outdoor experiments were conducted, and the temperatures were at −2°C. The number of failures was eight and six on the 4th and 5th of January, respectively. There was a notable difference in performance compared to indoor experiments. It is hypothesized that fewer failures would have been observed given warmer weather. It was noted that occasionally the display turned greenish after extended exposure to cold weather. Moreover, cold weather has the capacity to impact battery life and phone performance. Therefore, during the other experiments, the smartphone was connected to a power source in an effort to heat the battery and mitigate the cold weather’s effect on performance. Note the deterioration in the screen’s display in Figure 5.

**Figure 6:**
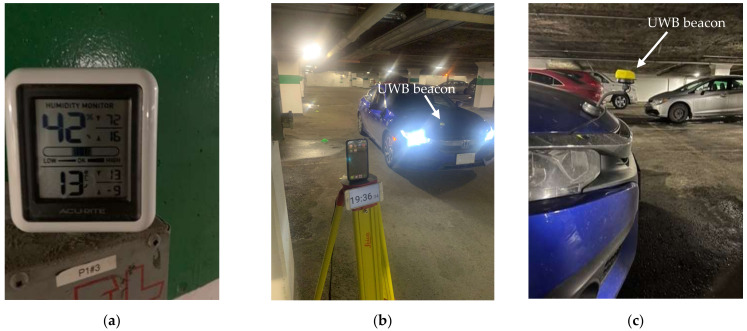
Indoor experimental setup showing (**a**) weather conditions, (**b**) a mounted phone and an approaching vehicle, and (**c**) a beacon mounted on the front right part of the vehicle.

The indoor experiments were carried out to simulate the performance under mild environmental conditions. Two cameras were placed to record the experiment inside the car to monitor the vehicle’s speed as it approached the phone’s location, as well as an externally fixed camera to monitor the phone during the hazardous vehicle approach. The temperature indoors was +13°C, and reference points were placed, as colored cones, every 2.5 m to estimate the vehicle location, as shown in Figure 6. Furthermore, the phone was connected to a power source. The number of failures dropped dramatically to only one out of a total of 61 trials conducted indoors.

Table 4 show a sample of the raw data recorded using the UWB equipment. As shown, *TTC*, Modified *TTC*, Mixed *TTC*, average speed, and acceleration are calculated; the distance between the beacon and phone is recorded. A trial typically starts when the UWB is actively communicating with the phone; that is, when the distance between them is dynamically measured. Then, the car moves to the trial starting position, which is at a distance of 25.00 m from the phone. Subsequently, the car accelerates from that position up to various target speeds: 3, 5, 10, and 15 km/h. The driver tries to maintain this speed once it is reached but ceases to maintain it at a later moment as the vehicle gets closer to the phone location. This is performed whenever necessary to maintain safe driving conditions. Please note that in all trials, the precise phone location was laterally offset by approximately 1.75 m from the beacon in order to eliminate the risk of a direct collision. Table 4 show the data obtained from an indoor experiment at a sample trial (Trial number 5).

**Figure 7:**
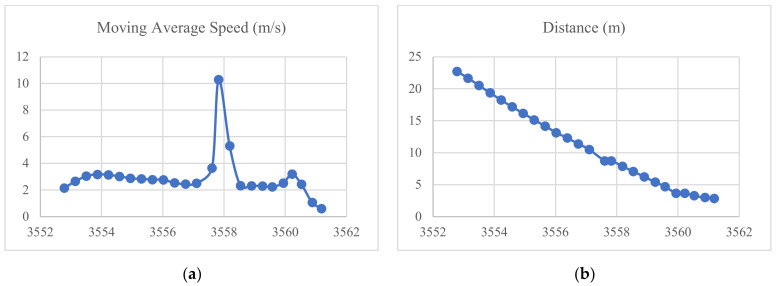

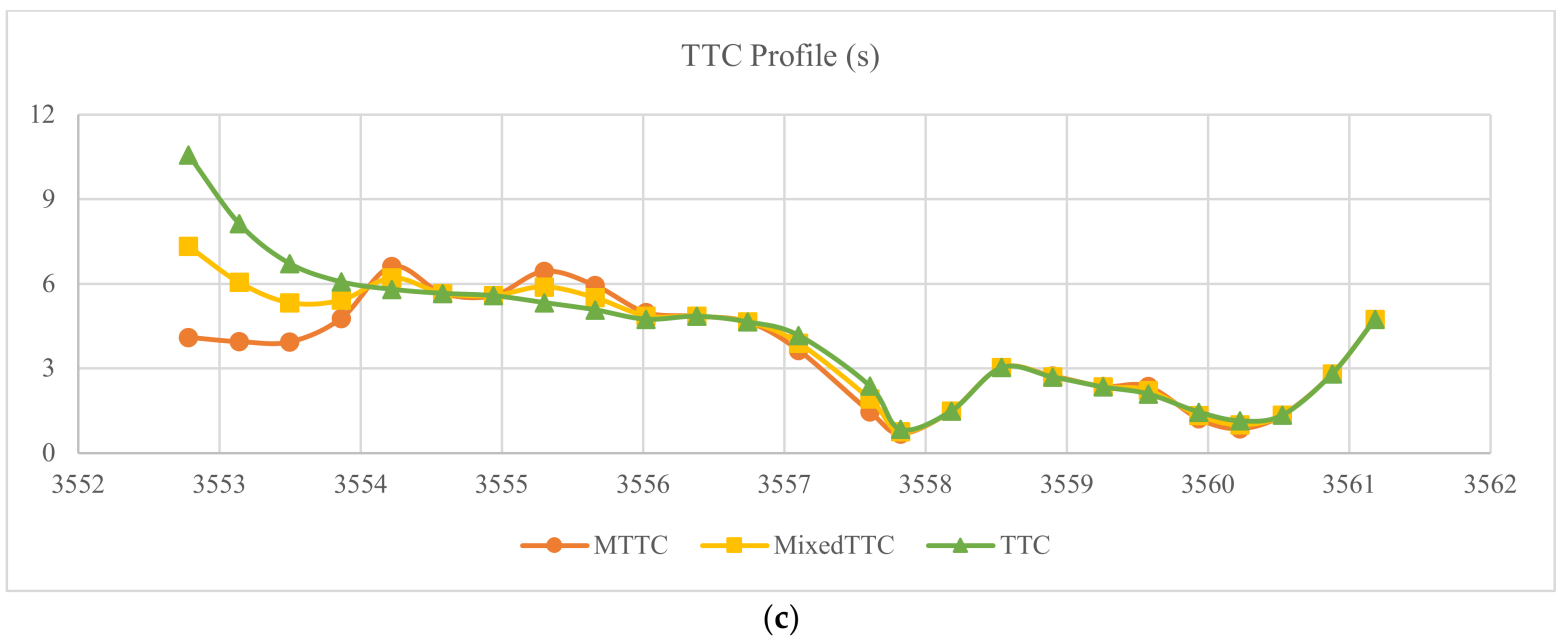
(**a**) Average speed over time. (**b**) Distance over time. (**c**) *TTC* profiles over time.

As shown in Table 4, Mixed *TTC* reached the critical level at 19(h):59(m):(s)17.603. Figure 7 shows the average speed over time, the distance over time, and the *TTC* profiles (one profile for each *TTC*) over time. It was noted that occasionally, when the notification is triggered, the average speed increases significantly immediately afterward because the application records another distance within approximately two milliseconds. This issue occurred randomly and unfortunately led to overestimating the post-trigger speed. It does not appear to influence the triggering mechanism in how it alerts the pedestrian or *TTC* measurements prior to the trigger. The exact reason for this issue was not clearly understood and requires further investigation.

**Figure 8:**
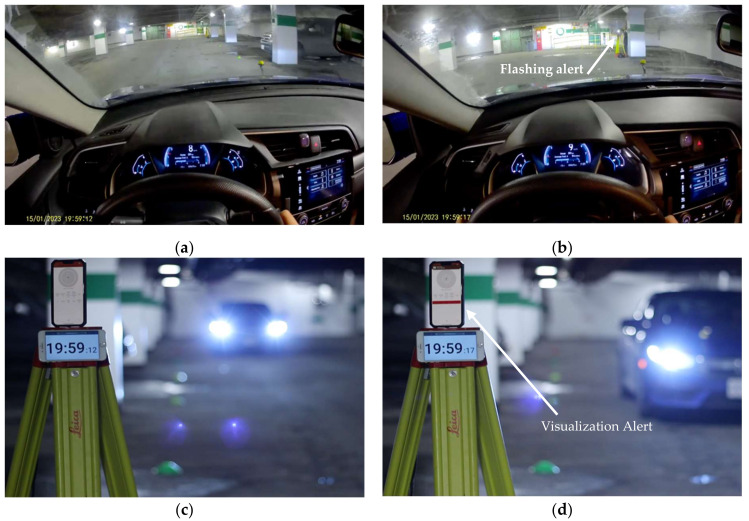
(**a**,**c**) Recordings of the camera view from inside the vehicle and from a roadside camera at times before and after the notification triggers. Notice the flash sent by phone in (**b**). Notice the color-coded notification in (**d**) at the same moment. Notice the synchronized clocks in both cameras.

**Figure 9:**
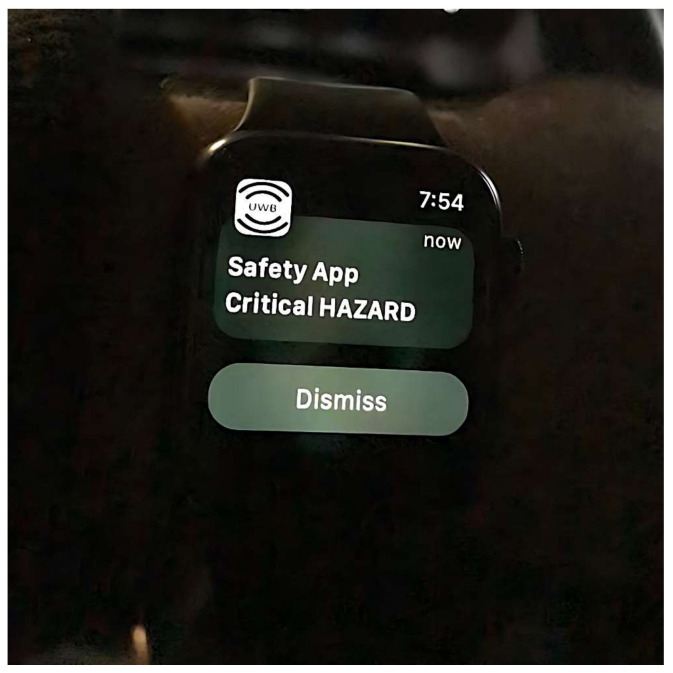
Sample notification sent to a connected smartwatch.

The real-time performance of this technology can be illustrated in Figure 8. For instance, the application detected a critical value of Mixed *TTC* at 19:59:17. The application changes the background color based on the level of risk. In this case, it was colored “orange,” indicating a medium risk level, or calculated the value of Mixed *TTC* in less than 2 s but greater than 1 s. The notification triggers an alert to the pedestrian by an alarm sound and sends a notification to a smartwatch with a message of “Critical Hazard,” as shown in Figure 9. Moreover, the phone flashes when Mixed *TTC* detects the risk of a collision to warn the driver of the potential hazard. 

### 3.2. Experiment Results

This section aims to provide an overview of the results and findings. The research looked at the average of different observations across all trials, as shown in Table 5. On average, the risk of collision was found to be triggered at various distances depending on the target vehicle speed. Specifically, the average distance at trigger time was 4.46 m at a speed of 3 km/h, 5.74 m at a speed of 5 km/h, 12.76 m at a speed of 10 km/h, and 15.44 m at a speed of 15 km/h. This is a reasonable performance, as the system detects a hazard further upstream in distance if the approaching vehicle is closing in faster.

A vehicle position marker (colored cones) was placed every 2.5 m on the right side of the travel path, as shown in Figure 8c. The longitudinal distance between the vehicle and phone at the moment of taking a given picture can be obtained from the camera by estimating the vehicle’s longitudinal location relative to the aforementioned distance markers. At the moment of trigger, the vehicle speed is observed by the internal camera placed inside the vehicle cabin, as shown in Figure 8b. The ground truth *TTC* at the alert trigger was calculated using vehicle information apparent in the cameras independent of UWB observations. This was performed for all trials at a speed of 10 km/h to represent average performance. As shown earlier, Figure 8d illustrates the estimated horizontal distance at trigger to be 7.5 m, and the vehicle speed appears to be 9 km/h (2.5 m/s) as shown in Figure 8b. The vehicle was laterally offset from the horizontal distance markers (cones) by 1.75 m, so the actual dynamic distances were calculated as shown in Table 6 as the oblique distance. Ground truth, or reported *TTC*, was calculated by dividing the actual distance by the vehicle speed obtained from the internal camera. Then, *TTC* measured using UWB equipment was compared to the reported *TTC* as shown in Table 6. The absolute mean difference between the two *TTC*s was 0.69 s at the moment of trigger.

The study further looked at the various classification performance measures such as sensitivity, specificity, and accuracy to evaluate the performance of UWB. Specificity and sensitivity were calculated based on the true positive and negative rates. True positive (TP) cases are when both the reported *TTC* and UWB *TTC* are less than the 3 s threshold. True negative (TN) cases are when both *TTC*s are greater than or equal to 3 s. False positive (FP) cases are when the reported *TTC* is greater than or equal to 3 s but the UWB *TTC* is less than 3 s. Finally, a false negative (FN) is when the UWB *TTC* is greater than or equal to 3 s, but the reported *TTC* is less than 3 s.

The true positive rate, also known as sensitivity, is defined as the portion of positive cases correctly identified as positive. Sensitivity can be calculated as: (TP)/(TP + FN). A highly sensitive classification algorithm will be associated with a high true positive rate. In this study, the classification algorithm calculated Mixed *TTC* and compared it to a threshold of 3 s. That will result in a small rate of failing to detect a positive case when it is truly positive. For example, it will be less likely to miss a case where *TTC* is less than 3 s when it is truly less than 3 s.

On the other hand, the true negative rate (TNR), also known as specificity, is the proportion of negative cases that are correctly identified as negative by the classification algorithm. Specificity can be calculated as (TN)/(TN + FP). Therefore, by evaluating the sensitivity and specificity of the performance of UWB, researchers and engineers can identify the performance of the UWB, discern the balance between positive and negative detection, and, hence, make enhancements for practical use. Finally, the accuracy of the UWB performance was calculated as follows:

Accuracy = (TP + TN)/(TP + FP + TN + FN).(3)

Sensitivity, specificity, and accuracy were initially calculated at the trigger moment. However, the sample size was small, and the performance measures were unstable. The reported *TTC* and UWB *TTC* were evaluated when the vehicle was observed to be precisely at an exact longitudinal distance, such as 20.00 m, 15.00 m, 10.00 m, and 5.00 m, for the indoor experiment. This is shown in Table 7 and Table 8. The UWB measurements were not recorded at precisely the same moment when the vehicle was adjacent to a cone. To address this issue, the corresponding UWB equipment measurements were calculated by taking the average of two detections. The times of those two detections were identified as follows: First, the timestamp at which the vehicle appears to be adjacent to a cone from the video cameras was recorded. This timestamp is to be read from the timing device apparent from the external camera. This is called the ground truth timestamp. Then, UWB measurements were adjusted to synchronize the timestamps with the timing device that appeared from the external camera by taking the average of two detections at each cone location. The two detections from UWB were identified by looking at the two closest timestamps before and after the ground truth timestamp.

The mean absolute differences between UWB measurements and ground truth measurements were calculated as shown in Table 7 and Table 8. In summary, the absolute mean differences between UWB measurements and ground truth data are 1.10 s, 0.88 m, and 0.46 m/s for *TTC*, distance, and speed, respectively. This is an important finding of those experiments that potentially lays the groundwork for future developments, knowing that the distance measurement error in mild weather conditions is approximately 0.88 m and the *TTC* measurement error is approximately 0.90 s. It is noteworthy that the ground truth distance is the oblique distance with a lateral offset of 1.75 m.

**Figure 10:**
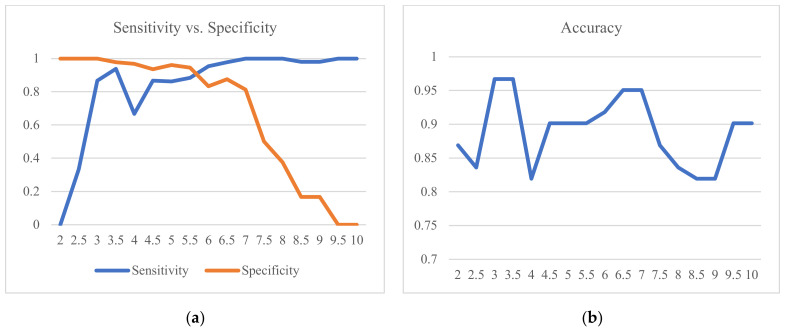
(**a**) Sensitivity vs. specificity over the *TTC* threshold. (**b**) The accuracy.

The threshold of 3 s was used in the experiment to classify events as either positive or negative. The threshold is used to construct the confusion matrix, which in turn significantly influences the sensitivity and specificity of UWB *TTC*. The choice of other thresholds should not materially affect the performance assessment. This was examined by recalculating the confusion matrices at other *TTC* thresholds and also recalculating the corresponding performance measures. Figure 10 illustrates the distribution of the sensitivity, specificity, and accuracy at various *TTC* thresholds. At a *TTC* of 3 s, the sensitivity, specificity, and accuracy were 0.87, 1.00, and 0.97, respectively, as shown in Table 9. Those are generally reasonable results that demonstrate a promising performance.

High specificity (100%) at the 3 s threshold means that there are no false positive detections. It indicates that the algorithm correctly identifies all non-collision cases and has a zero false positive rate. Generally, it is crucial to balance sensitivity and specificity to ensure the overall performance of estimating *TTC*. High specificity is crucial to minimize false positive detections, while high sensitivity is essential to ensure that potential or actual collisions are detected in a timely manner.

### 3.3. Smoothing Filters

**Figure 11:**
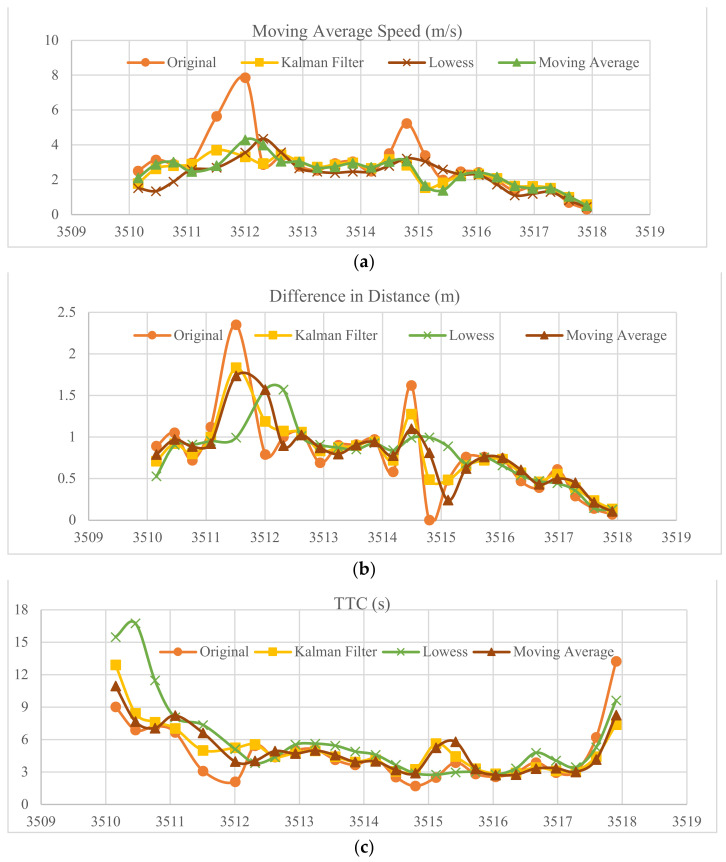
Smoothing filters used on (**a**) Average speed over time. (**b**) Difference in distance over time. (**c**) *TTC* profiles over time.

The distance detections were analyzed, and it was found that the difference in distance between every two detections is not consistent. For instance, Figure 11b shows the difference in distance from the UWB measurements. Delayed UWB measurements can potentially be caused by many reasons. First, the authors of [22,23] mentioned that UWB measurements can be impacted by environmental factors, such as surrounding obstacles, which leads to UWB measurements being influenced by multipath delays. Furthermore, the authors of [24] claimed that UWB measurements can be affected by errors in signal processing and that they can be potentially eliminated by adding multiple antennas. Therefore, further filtering processes should be applied in order to have accurate measurements.

Many filters were applied to the UWB distance measurements in order to overcome the issue of signal delays or errors in processing. The Kalman filter, moving average, and Locally Weighted Scatterplot Smoothing (Lowess) were applied to smooth UWB measurements. The Kalman filter, based on the expectation–maximization (EM) algorithm, was suggested by [25,26] to improve the estimation accuracy by calculating the parameters of the noise. The authors of [27] applied the Lowess filter to smooth data for pedestrian positioning systems. Figure 11 illustrates the performance of each filter. The Lowess filter was found to demonstrate a better result than the moving average and Kalman filters.

After applying the smoothing filters to the raw UWB distance measurements, the next step is validating the accuracy of the *TTC*. First, the *TTC* values were recalculated based on the type of smoothing filter used to process the distance measurements. The next step is to calculate the absolute mean difference of the *TTC* between the ground truth data from the video camera and smoothed UWB measurements. The following Table 10, Table 11, Table 12, Table 13 and Table 14 show that at a 20-meter distance, the absolute mean error of *TTC* was mostly over 2.00 s. However, the absolute mean error was 0.62 s, 0.81 s, 0.86 s, and 0.73 s for the raw UWB measurements and the Kalman, moving average, and Lowess filterings, respectively, excluding the absolute mean error at 20 m. Although the filters increased the mean error, the Lowess showed better results than the Kalman and moving average filters.

## 4. Discussion

The proposed UWB-based *TTC* estimation system has the potential to improve pedestrian safety. Moreover, the UWB technology demonstrates good performance and accuracy in calculating the location and speed of the moving ultra-wideband beacon. The proposed system has the capacity to alert a pedestrian in advance if there is a possibility of a collision. However, the UWB beacons used in this study required frequent changes of batteries (lithium disposable batteries), which indicates the need for a permanent power source if installed on vehicles. Furthermore, UWB signals have a relatively limited range, potentially limiting their use. After extensive testing, it appears that a maximum practical range of 25 meters is possible. This range may decline further to 20 m during cold weather conditions. The UWB beacon’s battery performance can be affected by cold weather, which results in reduced capacity and lifespan. More crucially, the iPhone’s U1 chip’s performance was found to be impacted by exposure to cold weather. The manufacturers need to note that and deploy more robust UWB chips that can perform more reliably in cold weather.

Simultaneous communication between the phone and two beacons was attempted. Unfortunately, it was found that this introduced a bottleneck in the measurement; that is, the phone was not able to independently record the distances for the two beacons and appeared to be restricted in this capacity. Therefore, the use of two beacons installed on the approaching vehicle was not associated with performance improvements. 

The presented technology focused mainly on measuring *TTC* and its variants. This was in line with the literature, which considers this the main collision indicator. Furthermore, *TTC* relies on predicted movements and hence can forecast hazards. Other indicators, e.g., PET, require the passage of time between both the pedestrian and vehicle at the same point or conflict area. It cannot forecast movement but studies actual movement, even if it includes evasive actions that alter and reduce the hazard levels. The precise way to calculate *TTC* has a significant impact on the safety assessment. It is paramount to design a robust technique to measure *TTC* that balances accuracy with robustness.

The developed prototype can potentially be used to alert a pedestrian to the danger of an approaching vehicle if the latter is too fast and/or too close. Furthermore, if connectivity between the phone and vehicle is possible, then the driver may be alerted to a nearby pedestrian who is being approached in an unsafe way. However, the testing of the developed prototype did not consider such cases, as the phone was static and mounted on a tripod, and the only notification visible to the driver was a flash from the camera light.

The technology needs to consider improving the stability of some detections, including after the notification trigger. Specifically, the post-trigger inaccuracy in speed measurement needs to be further investigated. Even though the UWB measurements were smoothed by using the Kalman, moving average, and Lowess filters, the accuracy may still be impacted by many factors, such as multipath effects and environmental interferences. The line-of-sight condition appeared to have an impact on distance measurements. The effect was complex because the distance detection frequency per minute appeared to drop in non-line-of-sight conditions. Therefore, further research and development are crucial to improving and providing better accuracy and more robust system performance in various environmental scenarios and conditions.

## 5. Conclusions

The presented research looked at the performance of UWB beacons under various environmental conditions. Strengths and limitations were discussed to inform the development of a new application to improve the performance of UWB beacons. An iOS application was used to calculate traffic conflict measures such as time-to-collision (*TTC*) and a customized variant of *TTC* was proposed in this study called Mixed *TTC*. The safety application can calculate the acceleration, moving average speed, distance, *TTC*, Modified *TTC*, and Mixed *TTC*. A notification alarm activates when the Mixed *TTC* value is at risk of collision, which is between 0 and 3 s. This alert includes a flash to warn the driver of potential hazards and a sound alarm to inform pedestrians of a critical hazard. Sensitivity, specificity, and accuracy were calculated to evaluate the performance of UWB. The Kalman, moving average, and Lowess filters were applied. The Lowess filter showed better smooth filtering than the Kalman and moving average filters. More studies can be performed involving various road users at signalized intersections or roundabouts with different smoothing filters to improve accuracy. Possible future enhancements may involve the integration of other sensor modes, including LiDAR and cameras, to improve the overall accuracy and reliability of the system. A limitation of this study is that it did not consider a representative sample of commercially available smartphones. Due to equipment compatibility at the time of this study, only one iPhone device was used. Another limitation of this study was that it only considered clear line-of-sight situations, which need to be further investigated when various objects can be present between the beacon and phone. Another limitation is that the study did not consider the effect of hot weather or extreme humidity on the performance of the technology. 

## Figures and Tables

**Table 1:** Sample of raw data for distance measurements.

Fingerprint	Date and Time	Beacon Estimated Distance (m)	Actual Distance (m)
Beacon 1	2022-12-26_09:54:04-888	1.01	1.00
Beacon 1	2022-12-26_09:54:05-193	1.1	1.00
Beacon 1	2022-12-26_09:54:05-506	1.1	1.00
Beacon 1	2022-12-26_09:54:05-812	1.13	1.00
Beacon 1	2022-12-26_09:54:06-116	1.13	1.00
Beacon 1	2022-12-26_09:54:06-427	1.11	1.00
Beacon 1	2022-12-26_09:54:06-728	1.1	1.00
Beacon 1	2022-12-26_09:54:07-043	1.12	1.00
Beacon 1	2022-12-26_09:54:07-356	1.11	1.00
Beacon 1	2022-12-26_09:54:07-673	1.12	1.00

**Table 2:** A sample of raw data for distance measurements.

Actual Distance (m)	Average UWB Beacon Distance (m)	MAPE (%)	MAPE (m)	Number of Detections per Minute
1.00	1.10	10.93	0.10	178
3.00	2.99	0.40	0.01	194
10.00	9.97	0.24	0.03	194
15.00	14.99	0.15	0.01	196
20.00	19.99	0.08	0.01	197
25.00	25.05	0.22	0.05	49
Average		2.00	0.03	168

**Table 3:** Number of trials at various speed limits and in indoor/outdoor settings.

Speed (km/h)	Outdoor	Failures	Indoor	Failures
3	15	3	15	1
5	34	9	16	0
10	31	1	15	0
15	25	3	15	0
Total	105	16	61	1

**Table 4:** A sample of raw data recorded in the safety application during a sample trial.

Trial 5								
Time	$\mathrm{Acceleration}\;(m/s^{2})$	Moving Average Speed (m/s)	Distance (m)	Modified Time-to-Collision (s)	Mixed Time-to-Collision (s)	Previous Speed (m/s)	Spot Speed (m/s)	Time-to-Collision (s)
19:59:12-418	1.59	1.55	23.58	4.55	9.87	1.22	1.89	15.19
19:59:12-778	1.65	2.15	22.72	4.11	7.34	1.89	2.41	10.58
19:59:13-137	1.43	2.66	21.67	3.96	6.06	2.41	2.91	8.15
19:59:13-494	1.10	3.05	20.53	3.94	5.34	2.91	3.19	6.73
19:59:13-861	0.36	3.18	19.37	4.78	5.43	3.19	3.18	6.08
19:59:14-218	−0.12	3.14	18.26	6.62	6.22	3.18	3.11	5.81
19:59:14-578	−0.31	3.03	17.19	5.67	5.67	3.11	2.96	5.67
19:59:14-938	−0.38	2.89	16.17	5.59	5.59	2.96	2.83	5.59
19:59:15-297	−0.15	2.84	15.15	6.45	5.90	2.83	2.85	5.34
19:59:15-658	−0.14	2.79	14.17	5.95	5.52	2.85	2.73	5.08
19:59:16-018	−0.05	2.77	13.15	4.99	4.87	2.73	2.81	4.75
19:59:16-378	−0.64	2.54	12.34	4.86	4.86	2.81	2.27	4.86
19:59:16-738	−0.26	2.45	11.39	4.66	4.66	2.27	2.62	4.66
19:59:17-098	0.20	2.52	10.52	3.65	3.91	2.62	2.42	4.18
19:59:17-603	3.15	3.66	8.75	1.47	1.93	2.42	4.90	2.39
19:59:17-820	7.92	10.29	8.75	0.68	0.76	12.28	8.29	0.85
19:59:18-180	−13.78	5.32	7.90	1.49	1.49	8.29	2.35	1.49
19:59:18-535	−8.41	2.33	7.08	3.04	3.04	2.35	2.31	3.04
19:59:18-896	−0.03	2.32	6.25	2.74	2.72	2.31	2.33	2.70
19:59:19-254	−0.02	2.31	5.42	2.37	2.36	2.33	2.29	2.35
19:59:19-574	−0.21	2.24	4.72	2.37	2.23	2.29	2.19	2.10
19:59:19-932	0.80	2.53	3.69	1.22	1.34	2.19	2.87	1.46
19:59:20-222	2.33	3.20	3.69	0.87	1.01	2.87	3.54	1.15
19:59:20-524	−2.54	2.44	3.29	1.35	1.35	3.54	1.33	1.35
19:59:20-877	−3.88	1.07	3.01	2.81	2.81	1.33	0.81	2.81
19:59:21-181	−1.52	0.61	2.88	4.75	4.75	0.81	0.41	4.75
19:59:21-489	−1.35	0.19	2.89	15.22	15.22	0.41	−0.03	15.22

**Table 5:** Average measurements from all trials were recorded at notification alert.

Approaching Vehicle Target Speed (km/h)	$\mathrm{Acceleration}\;(m/s^{2})$	Moving Average Speed (m/s)	Distance (m)	Modified Time-to-Collision (s)	Mixed Time-to-Collision (s)	Previous Speed (m/s)	Spot Speed (m/s)	Time-to-Collision (s)
3	2.05	1.57	4.46	1.74	2.44	1.06	2.08	3.14
5	1.93	1.98	5.74	1.95	2.56	1.61	2.35	3.16
10	3.17	3.77	12.76	1.96	2.65	3.02	4.52	3.35
15	3.55	4.92	15.44	1.91	2.53	4.52	5.33	3.15

**Table 6:** The estimated distances, speeds, and *TTC* from the cameras at the moment of trigger.

Trial	Speed (m/s)	Longitudinal Distance (m)	Oblique Distance (m)	Estimated *TTC* from Cameras (s)	*TTC* from UWB Beacon (s)	Absolute Difference (s)
1	2.50	7.5	7.70	3.08	3.41	0.33
2	2.77	7.5	7.70	2.78	3.13	0.35
3	2.77	7.0	7.22	2.60	3.09	0.49
4	3.33	15	15.10	4.54	3.10	1.44
5	2.50	7.5	7.70	3.08	2.39	0.69
6	3.05	12.5	12.62	4.14	3.61	0.53
7	2.77	7.5	7.70	2.78	3.32	0.54
8	2.77	7.0	7.22	2.60	3.46	0.86
9	2.77	8.0	8.19	2.96	3.25	0.29
10	2.77	8.0	8.19	2.96	3.23	0.27
11	2.77	7.0	7.22	2.60	2.02	0.58
12	2.77	17.5	17.59	6.35	3.79	2.56
13	2.77	8.0	8.19	2.96	3.11	0.15
14	2.77	8.0	8.19	2.96	3.51	0.55
15	2.77	12.5	12.62	4.56	3.84	0.72

**Table 7:** The estimated distances, speeds, and *TTC* from cameras and the UWB beacon from Trials 1 to 8.

	UWB Beacon Measurements			Ground Truth Data from Camera			Absolute Difference in Distance (m)	Absolute Difference in Speed (m/s)	Absolute Difference in *TTC* (s)
Trial	Moving Average Speed (m/s)	Distance (m)	*TTC* (s)	Moving Average Speed (m/s)	Distance (m)	*TTC* (s)			
1	2.910	21.030	7.260	1.94	20	10.29	0.95	0.96	3.03
	2.920	16.025	5.505	3.33	15	4.50	0.92	0.41	1.01
	2.975	11.525	3.880	3.06	10	3.27	1.37	0.08	0.61
	2.110	5.840	2.775	2.50	5	2.00	0.54	0.39	0.78
2	2.295	20.585	9.015	1.39	20	14.40	0.51	0.91	5.39
	2.700	15.920	5.920	2.50	15	6.00	0.82	0.20	0.08
	2.835	12.645	4.490	2.78	10	3.60	2.49	0.06	0.89
	2.745	5.800	2.130	2.78	5	1.80	0.50	0.03	0.33
3	2.660	21.315	8.385	2.22	20	9.00	1.24	0.44	0.62
	2.395	15.710	6.575	2.78	15	5.40	0.61	0.38	1.18
	2.545	11.505	4.595	2.78	10	3.60	1.35	0.23	1.00
	3.170	7.975	2.575	2.50	5	2.00	2.68	0.67	0.58
4	3.025	21.310	7.060	2.22	20	9.00	1.23	0.80	1.94
	5.370	16.190	3.785	3.33	15	4.50	1.09	2.04	0.72
	2.995	11.655	3.905	2.78	10	3.60	1.50	0.22	0.31
	2.450	6.600	2.700	2.50	5	2.00	1.30	0.05	0.70
5	2.855	21.100	7.440	2.22	20	9.00	1.02	0.63	1.56
	2.865	15.660	5.465	3.06	15	4.91	0.56	0.19	0.56
	2.495	11.865	4.760	2.78	10	3.60	1.71	0.28	1.16
	2.315	5.835	2.525	2.50	5	2.00	0.54	0.19	0.53
6	3.210	20.425	7.185	1.39	20	14.40	0.35	1.82	7.22
	2.900	15.380	5.345	3.06	15	4.91	0.28	0.16	0.44
	2.480	10.595	4.355	2.78	10	3.60	0.44	0.30	0.76
	2.245	5.965	2.670	2.78	5	1.80	0.67	0.53	0.87
7	2.405	22.130	9.210	2.50	20	8.00	2.05	0.09	1.21
	2.680	15.125	5.700	2.78	15	5.40	0.02	0.10	0.30
	2.620	11.765	4.570	2.78	10	3.60	1.61	0.16	0.97
	1.860	5.735	3.140	2.78	5	1.80	0.44	0.92	1.34
8	2.640	20.815	8.090	1.94	20	10.33	0.74	0.70	2.20
	2.715	15.415	5.675	2.78	15	5.44	0.31	0.06	0.27
	2.765	10.970	4.020	2.78	10	3.65	0.82	0.01	0.42
	2.325	5.555	2.420	2.78	5	1.91	0.26	0.45	0.62

**Table 8:** The estimated distances, speeds, and *TTC* from cameras and the UWB beacon from Trials 9 to 15.

	UWB Beacon Measurements			Ground Truth Data from Camera			Absolute Difference in Distance (m)	Absolute Difference in Speed (m/s)	Absolute Difference in *TTC* (s)
Trial	Moving Average Speed (m/s)	Distance (m)	*TTC* (s)	Moving Average Speed (m/s)	Distance (m)	*TTC* (s)			
9	2.905	21.705	7.705	2.50	20	8.03	1.63	0.41	0.30
	3.050	15.775	5.215	3.06	15	4.94	0.67	0.01	0.31
	2.515	11.770	4.750	2.78	10	3.65	1.62	0.26	1.15
	2.310	5.640	2.465	2.78	5	1.91	0.34	0.47	0.67
10	3.320	20.745	6.530	2.22	20	9.03	0.67	1.10	2.47
	3.070	15.800	5.185	2.78	15	5.44	0.70	0.29	0.22
	2.815	11.660	4.145	2.78	10	3.65	1.51	0.04	0.54
	2.460	5.855	2.410	2.78	5	1.91	0.56	0.32	0.61
11	3.710	20.655	5.715	1.94	20	10.33	0.58	1.77	4.57
	3.045	15.805	5.260	3.06	15	4.94	0.70	0.01	0.35
	2.580	11.295	4.415	2.78	10	3.65	1.14	0.20	0.82
	2.435	5.965	2.490	2.78	5	1.91	0.67	0.34	0.69
12	4.980	21.075	4.320	2.50	20	8.03	1.00	2.48	3.68
	2.650	16.250	6.215	2.78	15	5.44	1.15	0.13	0.82
	3.510	10.735	3.140	2.78	10	3.65	0.58	0.73	0.46
	1.830	5.555	3.050	2.50	5	2.12	0.26	0.67	1.05
13	2.295	21.365	9.320	2.22	20	9.03	1.29	0.07	0.32
	2.445	15.670	6.420	2.50	15	6.04	0.57	0.06	0.42
	3.080	11.245	3.715	2.78	10	3.65	1.09	0.30	0.12
	2.245	6.085	2.715	2.78	5	1.91	0.79	0.53	0.92
14	2.855	20.975	7.370	2.22	20	9.03	0.90	0.63	1.63
	2.995	15.780	5.275	2.78	15	5.44	0.68	0.22	0.13
	2.695	10.940	4.120	2.78	10	3.65	0.79	0.08	0.52
	2.260	5.645	2.515	2.50	5	2.12	0.35	0.24	0.52
15	2.620	20.620	8.110	1.94	20	10.33	0.54	0.68	2.18
	3.100	15.155	5.155	2.78	15	5.44	0.05	0.32	0.25
	3.045	11.110	3.940	2.78	10	3.65	0.96	0.27	0.34
	2.200	5.515	2.510	2.50	5	2.12	0.22	0.30	0.51
The mean absolute differences							0.88	0.46	1.1

**Table 9:** Classification table for the reported *TTC* and UWB *TTC*.

		Reported *TTC*	
		<3 s	>3 s
UWB *TTC*	<3 s	13	0
	>3 s	2	46

**Table 10:** Absolute mean difference of *TTC* (raw UWB measurements).

Difference in *TTC* at Each Distance		20 m	15 m	10 m	5 m
UWB Measurements	Trial 1	3.03	1.01	0.61	0.78
	Trial 2	5.39	0.08	0.89	0.33
	Trial 3	0.62	1.18	1.00	0.58
	Trial 4	1.94	0.72	0.31	0.70
	Trial 5	1.56	0.56	1.16	0.53
	Trial 6	7.22	0.44	0.76	0.87
	Trial 7	1.21	0.30	0.97	1.34
	Trial 8	2.20	0.27	0.42	0.62
	Trial 9	0.30	0.31	1.15	0.67
	Trial 10	2.47	0.22	0.54	0.61
	Trial 11	4.57	0.35	0.82	0.69
	Trial 12	3.68	0.82	0.46	1.05
	Trial 13	0.32	0.42	0.12	0.92
	Trial 14	1.63	0.13	0.52	0.52
	Trial 15	2.18	0.25	0.34	0.51
Average error at each distance		2.55	0.47	0.67	0.71
Average Error Excluding Errors at a distance of 20 m = 0.62 s					

**Table 11:** Absolute mean difference of *TTC* (after applying the Kalman Filter).

Difference in *TTC* at Each Distance		20 m	15 m	10 m	5 m
Kalman Filter	Trial 1	2.22	0.83	0.92	0.78
	Trial 2	4.63	0.15	1.09	0.33
	Trial 3	0.49	1.11	1.14	0.58
	Trial 4	0.97	0.90	0.58	0.70
	Trial 5	0.23	0.66	0.96	0.53
	Trial 6	5.88	0.21	1.00	0.87
	Trial 7	1.58	2.50	1.16	1.34
	Trial 8	1.44	0.17	1.00	0.62
	Trial 9	0.94	0.32	1.30	0.67
	Trial 10	1.81	0.08	0.78	0.61
	Trial 11	4.21	0.60	0.88	0.69
	Trial 12	2.09	0.88	0.03	1.05
	Trial 13	1.35	0.50	0.72	0.92
	Trial 14	1.08	0.31	1.19	0.52
	Trial 15	1.08	0.66	0.44	0.51
Average error at each distance		2.00	0.66	0.88	0.88
Average Error Excluding Errors at a distance of 20 m = 0.81 s					

**Table 12:** Absolute mean difference of *TTC* (after applying the Moving Average Filter).

Difference in *TTC* at Each Distance		20 m	15 m	10 m	5 m
Moving Average	Trial 1	2.88	0.97	0.62	0.74
	Trial 2	5.43	0.70	0.94	0.30
	Trial 3	1.26	1.16	0.95	0.87
	Trial 4	1.93	0.45	0.35	0.74
	Trial 5	1.54	0.55	1.56	0.38
	Trial 6	6.44	0.35	0.96	0.86
	Trial 7	0.38	1.53	1.72	1.33
	Trial 8	2.48	0.33	2.35	0.60
	Trial 9	0.55	0.29	1.85	0.69
	Trial 10	2.57	0.22	1.15	0.53
	Trial 11	4.76	0.53	0.82	0.66
	Trial 12	1.07	0.65	0.56	0.86
	Trial 13	0.22	0.39	1.96	0.91
	Trial 14	1.56	0.09	2.40	0.51
	Trial 15	2.51	1.73	0.05	0.53
Average error at each distance		2.37	0.66	1.22	0.70
Average Error Excluding Errors at a distance of 20 m = 0.86 s					

**Table 13:** Absolute mean difference of *TTC* (after applying the Lowess Filter).

Difference in *TTC* at Each Distance		20 m	15 m	10 m	5 m
Lowess Filter	Trial 1	2.68	0.56	0.93	0.37
	Trial 2	5.81	0.45	1.00	0.55
	Trial 3	1.95	0.12	0.62	0.56
	Trial 4	0.78	0.40	1.16	1.06
	Trial 5	2.53	0.56	0.12	0.00
	Trial 6	8.80	0.39	0.08	1.64
	Trial 7	2.31	0.28	0.81	1.67
	Trial 8	2.05	0.31	0.06	1.59
	Trial 9	2.19	1.93	1.74	1.59
	Trial 10	2.62	0.09	0.30	0.54
	Trial 11	2.11	0.28	2.23	0.41
	Trial 12	2.84	0.22	0.53	1.18
	Trial 13	0.20	1.41	1.72	0.45
	Trial 14	1.19	0.36	0.29	0.61
	Trial 15	2.03	0.62	0.05	0.98
Average error at each distance		2.67	0.53	0.77	0.88
Average Error Excludig Errors at a distance of 20 m = 0.73 s					

**Table 14:** Comparison between the Mean Absolute Error in *TTC* for all types of Filtering used.

Mean Absolute Error of *TTC* at Each Distance	Original UWB Estimation	Kalman Filter	Moving Average	Lowess
20 m	2.55	2.00	2.37	2.67
15 m	0.47	0.66	0.66	0.53
10 m	0.67	0.88	1.22	0.77
5 m	0.71	0.88	0.7	0.88
Average Error Excluding Errors at 20 m	0.62	0.81	0.86	0.73

## Data Availability

The data presented in this study pertaining to the wireless sensor measurements are available on request from the corresponding author. The data are not publicly available due to equipment privacy and security reasons. Other data may not be shared due to privacy reasons.
